# Urinothorax Secondary to Pyelonephritis With Obstructing Ureteric Calculi: A Rare Cause of Pleural Effusion

**DOI:** 10.7759/cureus.111247

**Published:** 2026-06-21

**Authors:** Anshuman Panda, Navneeth Jayprakash, Prashant Bhatia, Ashutosh K Singh, Sneha Mahalpure

**Affiliations:** 1 Critical Care Medicine, Asian Institute of Medical Sciences, Faridabad, IND; 2 Critical Care Medicine, S. S. Bansal (SSB) Heart and Multispecialty Hospital, Faridabad, IND; 3 Acute Medicine, The Princess Alexandra Hospital NHS Trust, Harlow, GBR

**Keywords:** double j stent, dyspnea, hydronephrosis (hdn), obstructive pyelonephritis, pleural fluid-to-creatinine ratio, rare cause of pleural effusion, renal calculi, retroperitoneal urine leak, ureteric calculi, urinothorax

## Abstract

Urinothorax is a rare cause of pleural effusion resulting from the presence of urine in the pleural space due to disruption of the urinary tract or obstructive uropathy. Only a limited number of cases have been reported in the literature to date. It is an underrecognized condition frequently delayed in diagnosis when respiratory symptoms predominate over genitourinary complaints. Prompt recognition is essential, as management must be directed at the underlying uropathy rather than the pleural effusion itself.

We report a case of urinothorax secondary to pyelonephritis with obstructing ureteric calculi in a 30-year-old man who presented with progressive breathlessness and required admission to the intensive care unit. Admission arterial blood gas analysis demonstrated metabolic acidosis with inadequate respiratory compensation (pH 7.29, partial pressure of arterial carbon dioxide 38 mmHg, bicarbonate 17 mEq/L, partial pressure of arterial oxygen 62 mmHg, and lactate 3.2 mmol/L). Noncontrast computed tomography of the chest and abdomen demonstrated right-sided ureteric calculi with mild hydroureteronephrosis, perinephric fat stranding, and fluid collections extending from the perinephric and posterior pararenal spaces cranially toward the right pleural cavity, consistent with urinary extravasation. Pleural fluid analysis demonstrated markedly elevated urea (135 mg/dL) and creatinine (6.9 mg/dL), yielding a pleural fluid-to-serum creatinine ratio of 2.65, confirming the diagnosis of urinothorax. Pleural fluid pH was 7.25, consistent with the characteristically low pH seen in urinothorax. Both urine and pleural fluid cultures grew multidrug-resistant *Escherichia coli*, and the patient was treated with meropenem. Bilateral double-J stenting led to progressive recovery in renal function (serum creatinine 2.6 mg/dL on admission, improving to 1.5 mg/dL by day 4) and successful weaning from respiratory support.

This case highlights the importance of clinical suspicion for urinothorax in patients with unexplained unilateral pleural effusion in the context of urinary tract pathology and reinforces that early biochemical pleural fluid analysis can direct timely, appropriate management.

## Introduction

Urinothorax is defined by the presence of urine within the pleural cavity secondary to disruption of the urinary tract or obstructive uropathy [1]. It is a rare diagnosis; fewer than 100 cases have been reported in the literature since its first description by Corriere et al. [2] in 1968, and is frequently underrecognized because respiratory symptoms may predominate over genitourinary complaints, misdirecting the initial clinical assessment [1,3]. Recognized etiologies include obstructive uropathy, renal calculi, urinary tract infection, trauma, and urological interventions such as ureteroscopy or percutaneous nephrolithotomy [3,4].

The underlying pathophysiology involves urinary leakage into the retroperitoneal space with subsequent cranial migration toward the pleural cavity, either through diaphragmatic defects or via lymphatic channels under conditions of increased retroperitoneal pressure [4,5]. The resulting pleural effusion is typically unilateral and ipsilateral to the affected kidney [5,6].

Diagnosis demands a high index of clinical suspicion and is supported by biochemical analysis of pleural fluid. A pleural fluid-to-serum creatinine ratio greater than 1 is the most specific diagnostic criterion, reflecting the higher creatinine concentration of urine relative to serum [6,7]. However, this ratio must be interpreted alongside clinical and radiological findings rather than used as a standalone criterion, as values only marginally above 1 may be nonspecific [7,8]. Urinothorax is the only recognized cause of a low-pH transudative pleural effusion, a characteristic that may help narrow the differential diagnosis when pleural fluid biochemistry is assessed [8,9]. Cross-sectional imaging, particularly computed tomography, is valuable in identifying ureteric obstruction, hydronephrosis, and retroperitoneal fluid collections extending cranially toward the diaphragm [9,10]. Management is directed at relief of the underlying urinary obstruction; pleural drainage alone provides only temporary symptomatic relief [10,11].

We present a case of urinothorax secondary to obstructing ureteric calculi with associated pyelonephritis, highlighting the role of clinical suspicion, biochemical confirmation, and radiological correlation in establishing this diagnosis.

## Case presentation

A 30-year-old man with no prior documented urological history or symptoms presented with a five-day history of progressive breathlessness, nonproductive cough, and reduced oral intake. There was no preceding flank pain, hematuria, or dysuria. Due to worsening respiratory distress, the patient required admission to the intensive care unit (ICU) and was initiated on high-flow nasal oxygen therapy; however, dyspnea persisted.

Admission arterial blood gas (ABG) analysis demonstrated metabolic acidosis with inadequate respiratory compensation: pH 7.29, partial pressure of arterial carbon dioxide (PaCO₂) 38 mmHg, bicarbonate 17 mEq/L, partial pressure of arterial oxygen 62 mmHg, and lactate 3.2 mmol/L. The PaCO₂ of 38 mmHg in the context of a bicarbonate of 17 mEq/L represents failure of adequate respiratory compensation, as the expected compensatory PaCO₂ by Winters' formula would be approximately 33-35 mmHg [12].

Laboratory investigations revealed leukocytosis, elevated inflammatory markers, and acute kidney injury. Both urine and pleural fluid cultures subsequently grew multidrug-resistant (MDR) *Escherichia coli*, confirming microbiological concordance between the urinary and pleural compartments. The patient was commenced on broad-spectrum antibiotics on admission and subsequently rationalized to meropenem following sensitivity results. The full laboratory findings are summarized in Table 1, serial renal function is shown in Table 2, and ABG parameters are shown in Table 3.

**Table 1:** Pleural fluid and diagnostic laboratory parameters LDH: lactate dehydrogenase; MDR: multidrug-resistant

Parameter	Result	Reference range	Units
Total leukocyte count	24,300	4,000-11,000	cells/mm³
C-reactive protein	Elevated	<10	mg/L
Blood urea nitrogen	90	7-20	mg/dL
Serum creatinine (admission)	2.6	0.6-1.2	mg/dL
Serum protein	6.7	6.0-8.0	g/dL
Serum LDH	240	140-280	U/L
Urinalysis	Pyuria, bacteriuria	-	-
Urine culture	MDR *Escherichia coli*	No growth	-
Pleural fluid urea	135	Not established	mg/dL
Pleural fluid creatinine	6.9	Not established	mg/dL
Pleural fluid-to-serum creatinine ratio	2.65	<1	Ratio
Pleural fluid pH	7.25	>7.30	-
Pleural fluid protein	1.5	Not established	g/dL
Pleural fluid LDH	180	Not established	U/L
Pleural fluid glucose	95	Not established	mg/dL
Pleural fluid total nucleated cells	380	Not established	cells/µL
Pleural fluid differential	Neutrophil predominance	-	-
Pleural fluid culture	MDR *Escherichia coli*	No growth	-
Pleural fluid protein/serum protein ratio	0.22	<0.5 (transudate)	Ratio
Pleural fluid LDH/serum LDH ratio	0.75	<0.6 (transudate)	Ratio

**Table 2:** Serial renal function following DJ stenting Serial renal function demonstrating progressive improvement in blood urea nitrogen and serum creatinine following bilateral DJ stenting, consistent with resolution of obstructive nephropathy. Discharge renal function was not available, as the patient was transferred to ward-level care upon ICU stabilization, and a formal discharge summary was not retrievable for this report ICU: intensive care unit; DJ: double-J

Timepoint	Blood urea nitrogen (mg/dL)	Serum creatinine (mg/dL)
Admission	90	2.6
Day 2 post-DJ stenting	70	2.0
Day 4 post-DJ stenting	45	1.5

**Table 3:** Serial arterial blood gas parameters Serial ABG parameters demonstrating progressive resolution of metabolic acidosis, hypoxemia, and hyperlactatemia following DJ stenting and supportive management. Admission ABG confirms metabolic acidosis with inadequate respiratory compensation; expected compensatory PaCO₂ by Winters' formula was 33-35 mmHg against a measured value of 38 mmHg. Recovery ABG demonstrates near-complete normalization of acid-base status and oxygenation PaCO₂: partial pressure of arterial carbon dioxide; HCO₃⁻: bicarbonate; PaO₂: partial pressure of arterial oxygen; DJ: double-J

Parameter	Admission	Postintervention	Recovery
pH	7.29	7.35	7.40
PaCO₂ (mmHg)	38	34	38
HCO₃⁻ (mEq/L)	17	20	24
PaO₂ (mmHg)	62	85	95
Lactate (mmol/L)	3.2	1.8	1.2

Thoracic ultrasonography identified a right-sided pleural effusion. Noncontrast computed tomography (CT) of the chest and abdomen was subsequently performed; contrast administration was withheld given the degree of renal impairment (serum creatinine 2.6 mg/dL). Following CT, an intercostal chest drain was inserted for therapeutic drainage and diagnostic pleural fluid sampling.

**Figure 1:**
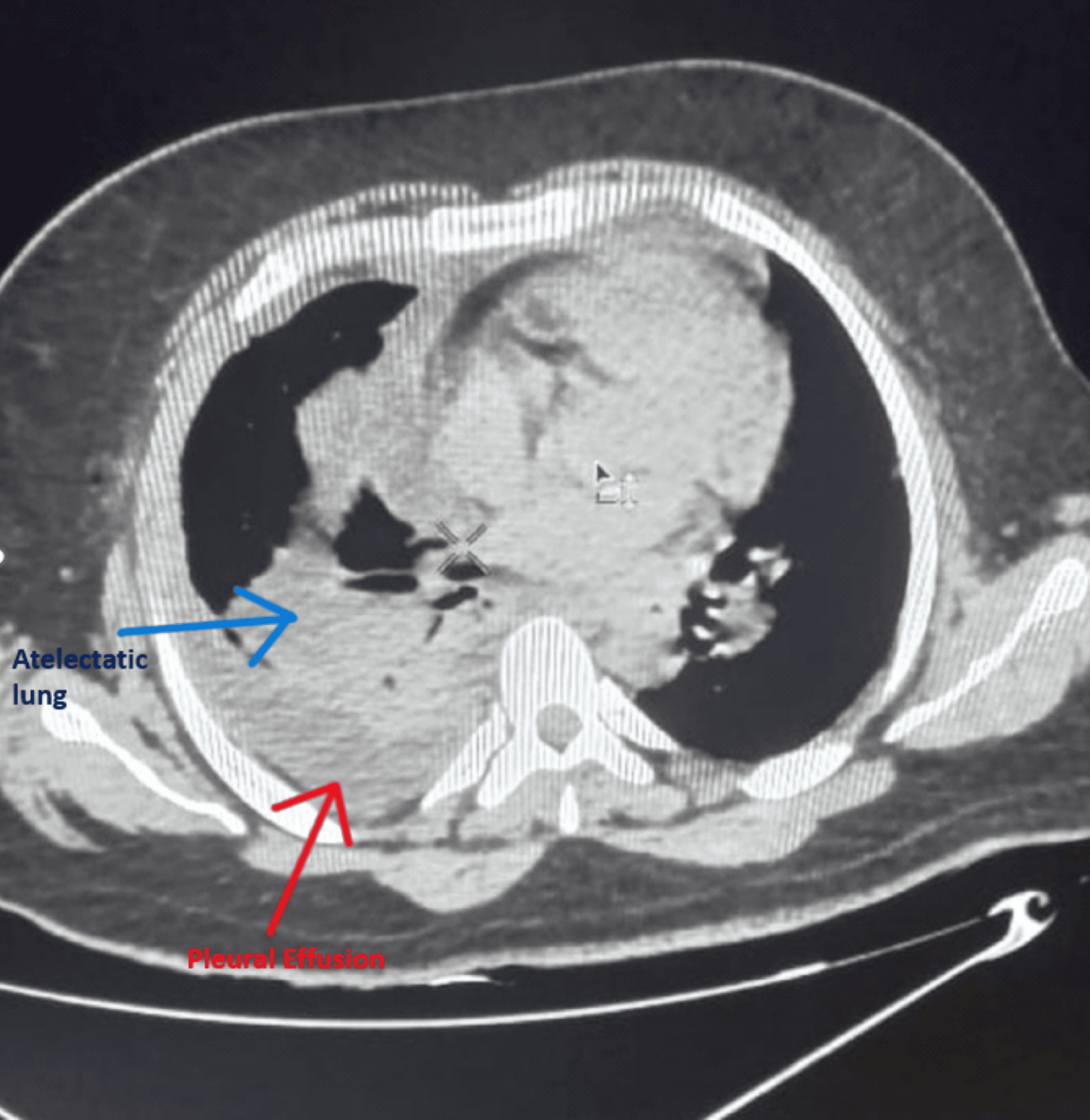
Axial CT chest demonstrating right-sided pleural effusion with associated pulmonary atelectasis Axial noncontrast CT chest demonstrating a right-sided pleural effusion (red arrow) with associated atelectasis of the underlying right lung parenchyma (blue arrow). The effusion occupies the right pleural space with passive collapse of the adjacent lung CT: computed tomography

**Figure 2:**
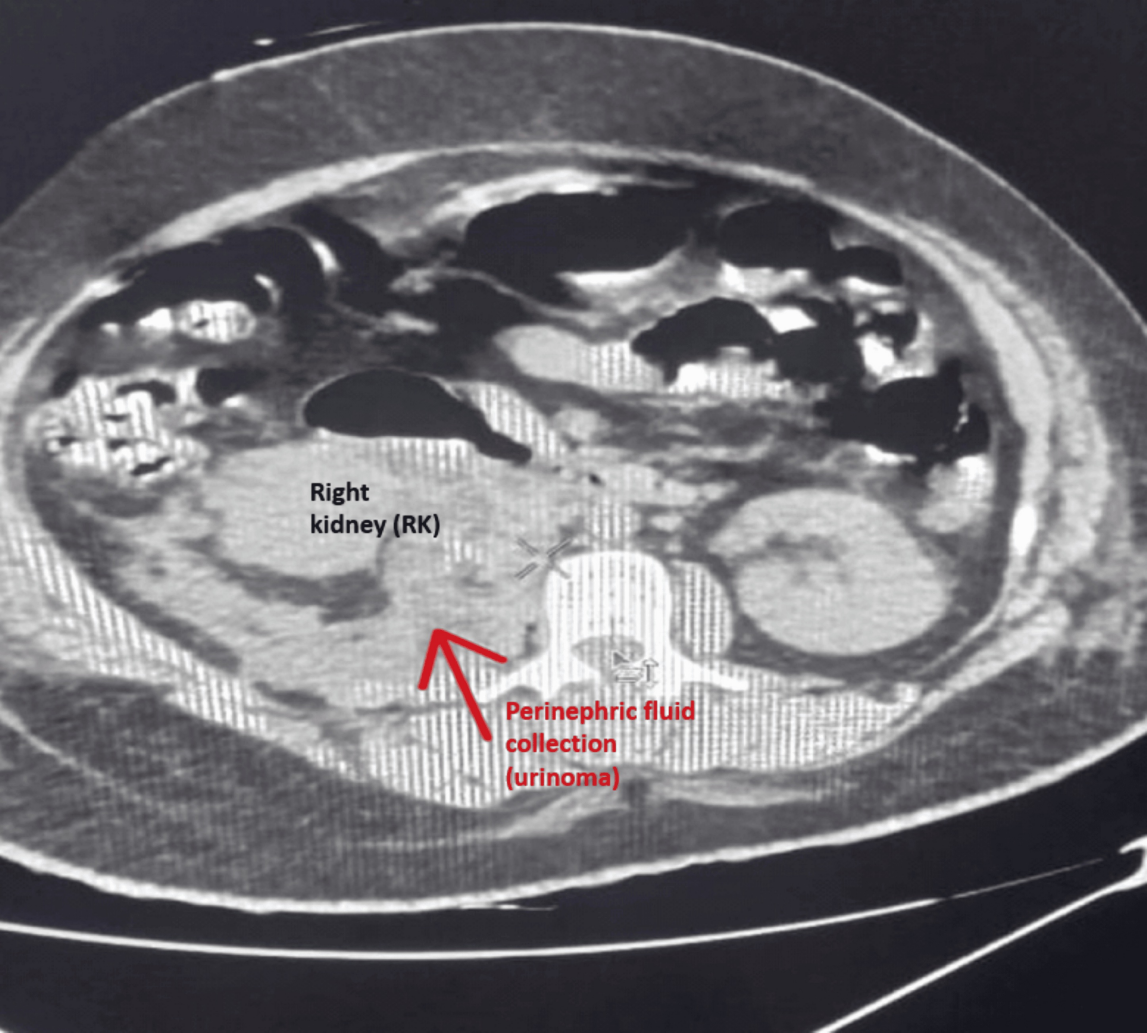
Axial CT abdomen demonstrating right perinephric fluid collection Axial noncontrast CT abdomen at the level of the renal hilum demonstrating a right-sided perinephric fluid collection (arrow) in the setting of obstructive uropathy secondary to ureteric calculi. The collection is situated within Gerota's fascia and is consistent with urinary extravasation forming a urinoma. The right kidney is visible to the right with mild hydroureteronephrosis CT: computed tomography

**Figure 3:**
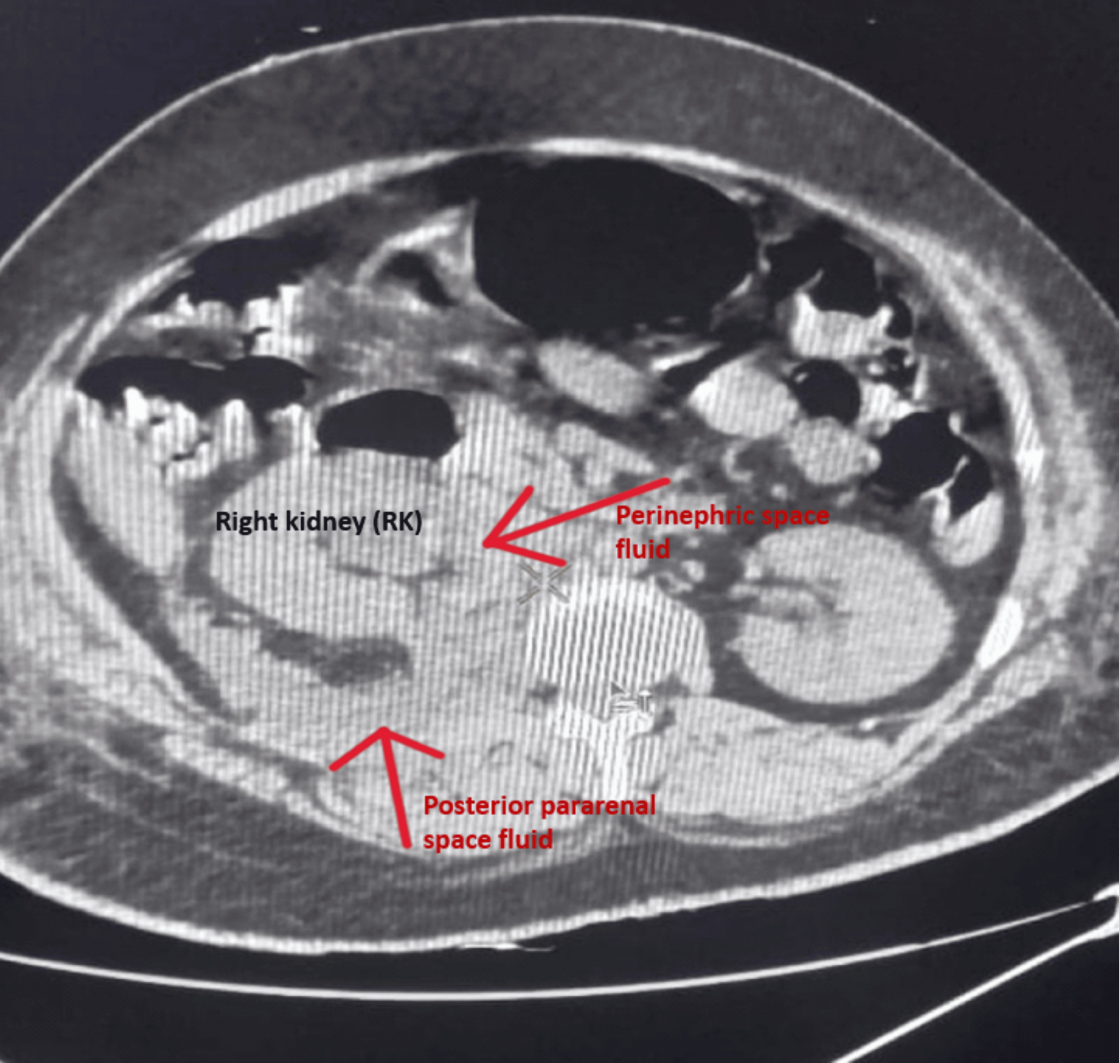
Axial CT abdomen demonstrating retroperitoneal fluid collections in the perinephric and posterior pararenal spaces Axial noncontrast CT abdomen demonstrating retroperitoneal fluid collections consistent with urinary extravasation. The upper arrow indicates fluid within the right perinephric space (between Gerota's fascia and the renal capsule), consistent with a urinoma. The lower arrow indicates extension of fluid into the posterior pararenal space. The right kidney is visible to the right of the midline with surrounding fat stranding CT: computed tomography

**Figure 4:**
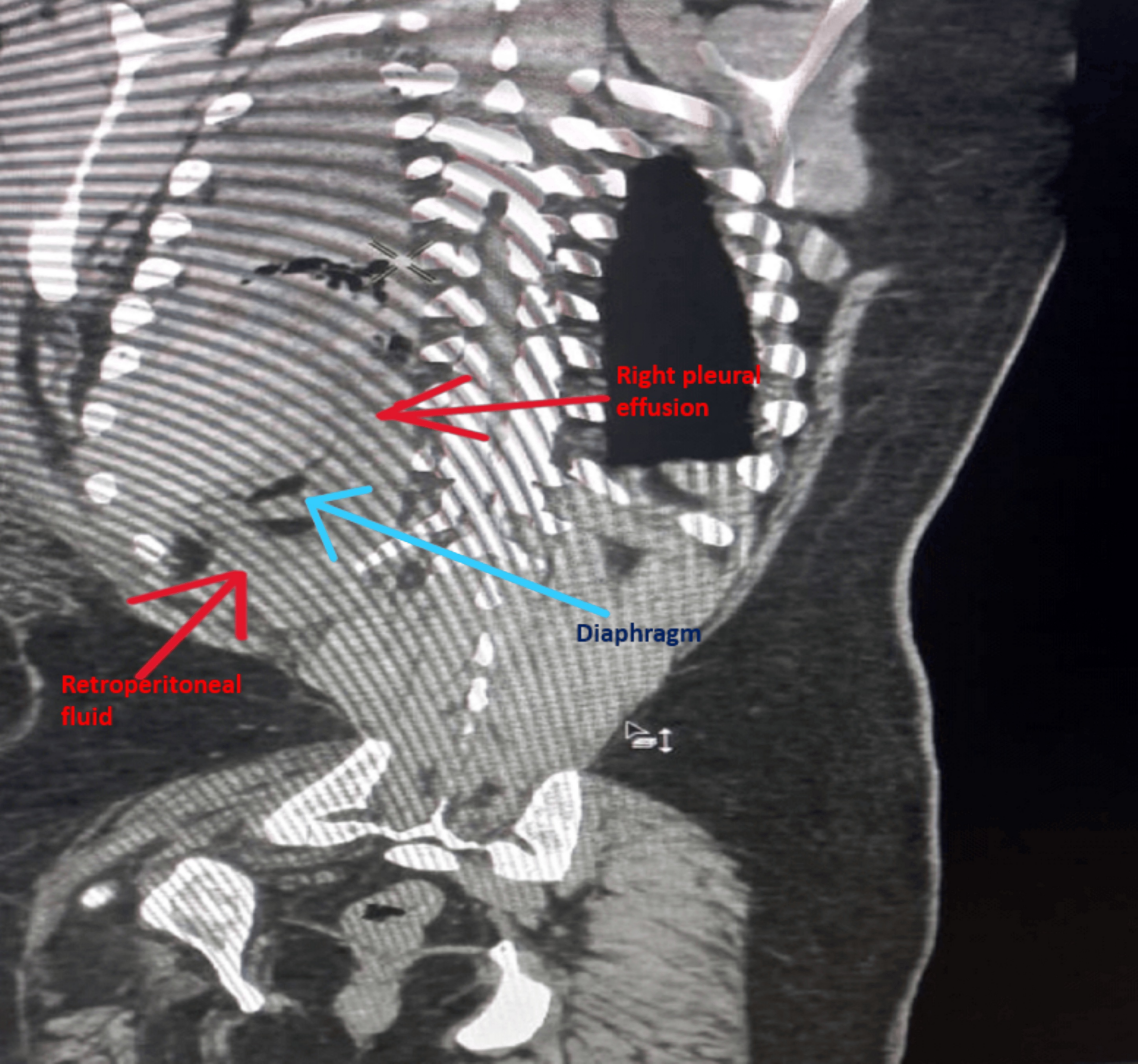
Coronal CT reconstruction demonstrating cranial extension of retroperitoneal fluid into the right pleural cavity Coronal noncontrast CT reconstruction demonstrating retroperitoneal fluid (lower arrow) extending cranially along retroperitoneal fascial planes toward and through the right hemidiaphragm, with associated right-sided pleural effusion (upper arrow). This imaging pattern supports suspected communication between the retroperitoneal space and the pleural cavity, consistent with urinothorax. The proposed mechanism includes either direct passage through a diaphragmatic defect or indirect migration via lymphatic channels under elevated retroperitoneal pressure CT: computed tomography

CT chest demonstrated a right-sided pleural effusion with associated atelectasis of the underlying right lung parenchyma (Figure 1). Abdominal CT demonstrated two calculi measuring 6 and 7 mm in the right upper ureter with mild hydroureteronephrosis, perinephric fat stranding, and renal fascial thickening, in keeping with obstructive pyelonephritis. Loculated fluid collections were identified in the right perinephric space (Figure 2), consistent with a urinoma arising from fornical rupture under obstructive pressure, with extension into the posterior pararenal space (Figure 3). Coronal reconstruction demonstrated cranial extension of these retroperitoneal fluid collections along fascial planes toward the right hemidiaphragm, with an associated right-sided pleural effusion (Figure 4), supporting a suspected communication between the retroperitoneal space and the pleural cavity via diaphragmatic defects or lymphatic channels.

The differential diagnoses considered at presentation included parapneumonic effusion, empyema, reactive pleural effusion secondary to pyelonephritis, and malignancy-related effusion. Parapneumonic effusion and empyema were made less likely by the absence of consolidation on imaging, the normal pleural fluid glucose (95 mg/dL), and the isolation of MDR *E. coli* in both pleural and urinary cultures rather than typical respiratory pathogens. Malignancy-related effusion was excluded by the patient's age, absence of any mass lesion or lymphadenopathy on CT, and the clinical context of obstructive uropathy. The combination of obstructive calculi, retroperitoneal fluid tracking cranially into the pleural cavity, a pleural fluid-to-serum creatinine ratio of 2.65, low pleural fluid pH, and microbiologically concordant cultures was consistent with urinothorax as the unifying diagnosis.

Urology was consulted, and the patient underwent bilateral double-J (DJ) stenting for relief of urinary obstruction. Despite initial pleural drainage and oxygen therapy, breathlessness and hypoxemia persisted. Repeat CT chest demonstrated a persistent moderate right-sided pleural effusion with encysted components and partial collapse of the underlying lung parenchyma with the pigtail catheter in situ. Cardiothoracic surgical consultation was obtained, and decortication was discussed; however, the patient was managed conservatively. Following DJ stenting and meropenem therapy, progressive clinical improvement ensued, with serial renal function showing a downward trend in creatinine from 2.6 to 1.5 mg/dL over four days (Table 2), and ABG parameters normalizing toward recovery (Table 3). The patient was successfully weaned off respiratory support and transferred to the ward upon ICU stabilization.

## Discussion

Urinothorax is a rare and underrecognized cause of pleural effusion, with fewer than 100 cases reported in the literature worldwide since its first description in 1968 [1,3]. The diagnosis is frequently delayed when respiratory symptoms dominate the clinical picture, as occurred in this case, where progressive breathlessness and hypoxemia initially suggested a primary respiratory or infective etiology [3].

The pathophysiology in obstructive urinothorax involves urinary extravasation into the retroperitoneal space under conditions of elevated intrarenal pressure, with subsequent cranial migration of fluid toward the pleural cavity either through diaphragmatic defects or via lymphatic channels [4,5]. The retroperitoneal fluid collections in this case, involving the perinephric and posterior pararenal spaces, are consistent with a urinoma arising from fornical rupture under obstructive pressure, which subsequently tracked along retroperitoneal fascial planes toward the pleural cavity [6]. Noncontrast CT was appropriately selected given the degree of renal impairment, and while this precluded direct visualization of contrast extravasation or delayed excretory-phase imaging, the combination of imaging morphology, fluid distribution, biochemical profile, and microbiological concordance provides convergent diagnostic evidence.

The pleural fluid biochemistry in this case is diagnostically robust. The pleural fluid-to-serum creatinine ratio of 2.65 is substantially above the diagnostic threshold of greater than 1, providing strong biochemical support for urinary origin of the effusion [7]. However, it is important to recognize the limitations of this marker when used in isolation. Creatinine equilibrates relatively freely between the pleural space and serum, and ratios only marginally elevated above 1 may not reliably discriminate urinothorax from other effusions [8]. Clinicians should, therefore, integrate the creatinine ratio with clinical context, imaging findings, and, as in this case, microbiological data.

A particularly noteworthy biochemical finding is the pleural fluid pH of 7.25. Urinothorax is recognized in the literature as the only cause of a low pH transudative effusion, and this characteristic, when present, is a valuable discriminating feature that can help narrow the differential diagnosis at the bedside [8,9]. Additionally, application of Light’s criteria in this case yields a discordant result [13]: the pleural fluid protein-to-serum protein ratio of 0.22 classifies the effusion as a transudate, while the lactate dehydrogenase (LDH) ratio of 0.75 meets the exudative threshold. This LDH-discordant exudate pattern in urinothorax is well described in the literature; the systematic review by Toubes et al. noted that a significant proportion of urinothorax cases classified by Light’s criteria were LDH-discordant exudates, and likely reflects local inflammatory and infectious processes elevating pleural LDH without a true exudative mechanism [1]. Clinicians should be aware that urinothorax may not fulfill classic transudative criteria by Light’s criteria alone, and that overreliance on this classification may lead to diagnostic misdirection.

A clinically significant finding unique to this case is the microbiological concordance between urine and pleural fluid cultures, both growing MDR *E. coli*. This directly supports true communication between the urinary and pleural compartments rather than independent pleural infection, and has direct therapeutic implications: targeted antimicrobial therapy active against the causative organism, in this case meropenem, is required alongside relief of obstruction. Infected urinothorax represents a particularly severe clinical phenotype and has been associated with worse outcomes when urinary obstruction is not promptly addressed [7].

Management of urinothorax must be directed at the underlying urinary obstruction [9,10]. Pleural drainage provides symptomatic relief but does not resolve the effusion unless the source of leakage is treated. In this case, bilateral DJ stenting achieved decompression of the obstructed system and was followed by a clear, progressive improvement in both renal function (creatinine 2.6 → 2.0 → 1.5 mg/dL over four days) and acid-base status and oxygenation on serial ABGs. The persistence of a moderate encysted effusion after stenting, requiring consideration of decortication, likely reflects the inflammatory sequelae of infected urinothorax, which may complicate the expected resolution seen in uncomplicated cases.

A key clinical learning point from this case is the importance of including pleural fluid creatinine, alongside pH, protein, LDH, and glucose, in the biochemical panel for any patient presenting with unexplained unilateral pleural effusion in the context of urinary tract pathology. This simple addition to routine pleural fluid analysis can rapidly establish the diagnosis and redirect management toward the urological cause.

Several limitations of this report should be acknowledged. Noncontrast CT, while clinically appropriate, precluded direct radiological confirmation of urinary leakage via contrast extravasation or excretory-phase imaging. Formal discharge renal function and long-term follow-up were unavailable, as the patient was transferred to ward-level care upon ICU stabilization without a retrievable discharge summary. Definitive stone management following the acute episode was not documented. These limitations are inherent to the retrospective single-center nature of this report.

## Conclusions

Urinothorax is a rare and underrecognized cause of pleural effusion that should be included in the differential diagnosis of any patient with unexplained unilateral pleural effusion in the context of obstructive uropathy, renal calculi, or urinary tract infection. This case demonstrates that the condition may present with predominant respiratory symptoms and metabolic derangement, and that early comprehensive pleural fluid biochemical analysis, including creatinine, pH, protein, LDH, and glucose, alongside cross-sectional imaging, can rapidly establish the diagnosis. A pleural fluid-to-serum creatinine ratio of 2.65, combined with a low pleural fluid pH of 7.25, an LDH-discordant exudate pattern by Light’s criteria, and microbiologically concordant MDR *E. coli* cultures in both urinary and pleural compartments, provided convergent diagnostic evidence in this case. Management directed at relieving urinary obstruction with bilateral DJ stenting, combined with targeted antimicrobial therapy and supportive care, resulted in progressive clinical, biochemical, and physiological recovery as demonstrated by serial renal function and ABG parameters. Increased awareness of this rare entity among clinicians in critical care, respiratory medicine, and urology is essential to facilitate timely diagnosis and appropriate multidisciplinary management.
